# Case Report: NET of the ear- up to date in diagnostic and therapeutic challenges

**DOI:** 10.3389/fonc.2026.1687587

**Published:** 2026-03-13

**Authors:** Giulia Puliani, Maria Flavia Bagaglini, Marta Bianchini, Antonietta Fasciglione, Rosa Lauretta, Marilda Mormando, Marialuisa Appetecchia

**Affiliations:** Oncological Endocrinology Unit, IRCCS Regina Elena National Cancer Institute, Rome, Italy

**Keywords:** diagnosis, MeNETS, NET of the middle ear, prognostic factors, treatment, neuroendocrine neoplasm

## Abstract

Neuroendocrine tumors (NET) usually originate from the gastro-entero-pancreatic or pulmonary tract. However, with the improvement of diagnostic techniques, the identification of NET arising in unusual origins is increasing, including the middle ear, but little is known about their management. We present three cases of NET of the middle ear (MeNET), with a special focus on histological diagnosis, radiological, and nuclear medicine imaging. We describe the surgical intervention performed and the systemic treatment used in one case of metastatic disease. We also conducted a review of the published cases, collecting data only on well-differentiated MeNET, histologically characterized by the presence of glandular/epithelial and neuroendocrine cells, with specific immunohistochemistry features (Cytokeratins+, Cromogranin A+, Synaptophysin +, enolase +, INSM1+). Our case series and literature review analysed the main clinical features, diagnostic challenges, and treatment response of this rare localization of NET, trying to provide a guide for the diagnosis and management of this rare disease.

## 1 Introduction

NET of the middle ear (MeNET) are very rare tumors arising from neuroendocrine cells in the middle ear (1). In the past, the terms “neuroendocrine adenoma”, “carcinoid”, “adenomatous neoplasm”, and “adenomatous neuroendocrine tumor” have been used as synonyms. However, considering histological details, not all these entities show the same histological pattern and natural history. The World Health Organization (WHO) 2022 neuroendocrine tumor classification clearly distinguished between neuroendocrine tumor (NET), neuroendocrine carcinoma (NEC), and mixed neuroendocrine tumor (2), and the WHO 2022 classification of head and neck tumors confirmed the need to use this neuroendocrine “universal” nomenclature (3) for this origin as well (4). In this study, we presented three cases of MeNET from our institution,. Furthermore, a narrative review of the existing literature was performed to summarize the current knowledge on the pathogenesis, diagnostic parameters, and therapeutic strategies associated with this rare neoplastic entity, focusing only on MeNETs.

## 2 Materials and methods

We presented a case series from our centre. Chromogranin A (CgA) and enolase (NSE) values were measured using standardized commercially available immunoassays routinely adopted at our Institution. The same analytical methods and laboratory reference ranges were applied consistently for each patient and during follow-up. The reference range for CgA was <100 U/L, while NSE values were <15 ng/ml. In addition, we performed a narrative review on PubMed database to identify clinical cases of MeNET published after January 2000, chosen for the first WHO classification of neuroendocrine tumors including tumor grade. The last search was performed in December 2025. Inclusion criteria were: English-written articles, on adult patients (over 18 years old) with a confirmed histological diagnosis of middle ear NET. In cases with unclear histology (such as “adenoma” or “adenomatous neuroendocrine tumor”), the histopathological data have been reassessed to include only cases that match the actual definition of MeNETs. Therefore, any article that did not meet these criteria —such as pediatric cases or reports lacking key immunohistochemical or histopathological details—was excluded. The string used was: (“middle ear”) AND (“neuroendocrine tumor” OR “neuroendocrine tumour” OR adenoma OR carcinoid OR adenomatous). Following the application of the aforementioned search criteria, a total of 79 articles were identified. A standardized data collection form was used for extracting these data: clinical presentation, radiological findings (either from computed tomography, CT, magnetic resonance imaging, MRI, or positron emission tomography- computed tomography, PET-CT scans), histopathological findings including immunohistochemical results, stage of the disease, treatment performed, duration of follow-up, recurrences and survival.

## 3 Case series

### 3.1 Case 1

A 30-year-old man came to our attention in 2012 for persistently high serum values of chromogranin-A (CgA) (>300 U/L), which raised the possibility of a neuroendocrine tumor (NET). The patient presented normal physical tests and no clinical symptoms. Conventional imaging was unable to detect the cause of the elevated biomarker. The patient, therefore, underwent a 68Ga-DOTANOC PET/CT that showed a focused area of enhanced radiopharmaceutical uptake (maximum standardized uptake value = 4.5) in the right middle ear. The lesion was surgically removed, and the histology revealed a well-differentiated NET (on immunohistochemical staining, tumor cells were positive for cytokeratin, chromogranin, and synaptophysin, Ki-67 labelling index (LI) was 2%, configuring grade 1 NET). The tumor was confined to the middle ear and showed no signs of metastasis or local invasion. Following the surgery, the CgA levels returned to normal, and follow-up with 68Ga-DOTANOC PET/CT after one year showed no recurrence of the tumor, indicating the patient was disease-free. The patient was monitored for 2 years without recurrence of the disease, and after this data, he was lost to follow-up. Genetic testing was not routinely performed at the time of diagnosis. This case was already reported (5).

### 3.2 Case 2

In November 2016, a 32-year-old woman reported pain in her left ear that would not let up. After several visits and unclear results on ear examination, a mastoid biopsy showed an epithelial cells growth with neuroendocrine features, confirming MeNET. Histological analysis revealed fibrovascular tissue with eosinophilic epithelial cells devoid of mitotic activity. CK8/18+, CK7±, CK20–, synaptophysin+, chromogranin±, and a Ki-67 LI <5% were all detected by immunohistochemistry. In December 2016, a 17 × 8 mm mass in the left mastoid with expansion into the epitympanum was identified on MRI, showing heterogeneous contrast enhancement. CT scans performed in March 2018 and March 2019 revealed increasing bone degradation and possible surgical alterations in the left otomastoid area. The ossicular chain was encapsulated in the lesion without any erosion. Somatostatin receptor expression was demonstrated by functional imaging using 111In-Octreoscan (July 2019) and 68Ga-DOTANOC PET/CT (June 2019), which revealed radiotracer uptake limited to the left mastoid.

Despite her young age, genetic testing was not performed. The patient was referred to our Center at the end of July 2019. CgA levels were found to be normal. A stable lesion in the left tympanic cavity with accompanying otomastoiditis and no signs of perineural spread was confirmed by MRI in December 2019. In November 2020, the patient underwent surgical intervention to remove the mass. Histopathological examination confirmed MeNET, with diffuse expression of synaptophysin, CD56, and cytokeratin, negative chromogranin, and a low Ki-67 LI (2%). No recurrence or metastasis was observed during the following 4 years of follow-up.

### 3.3 Case 3

**Figure 1:**
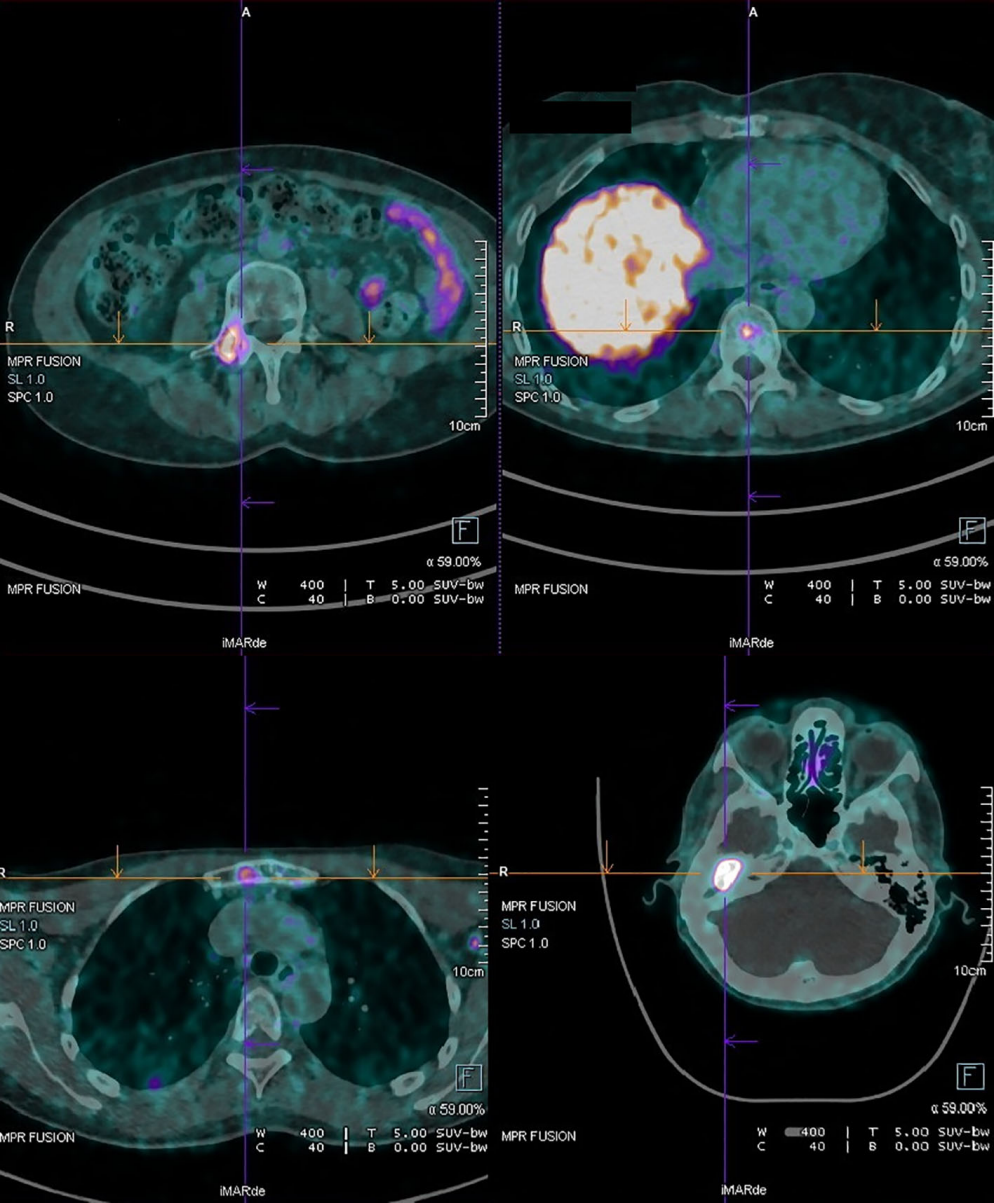
68Ga-DOTATATE PET/CT scan performed in July 2024 in case 3. It demonstrated an increase in the expression of somatostatin receptors in the right middle ear and skeletal sites (sternum, vertebrae).

In April 2023, a 54-year-old woman was referred to our Center for evaluation of a MeNET. Her symptoms started in 2019 with ongoing loss of hearing on the right side along with discharge from the ear, which progressively worsened and did not respond to antibiotics. The evaluation was also delayed because of the COVID-19 pandemic. In June 2022, a CT scan showed a mass in the right tympanic cavity, treated by tympanoplasty. Histopathological analysis confirmed it was a MeNET, and immunohistochemical staining was positive for cytokeratin, synaptophysin, chromogranin A, and enolase. Staining for p63 showed spot positivity; Ki-67 LI was less than 5%. CD56, CK7, and S100 stainings were negative. Results of a 68Ga-DOTATATE PET/CT performed in December 2022 showed radiotracer uptake in the right middle ear, along with multiple sites of suspected bone involvement (the skull, sternum, thoracic and lumbar vertebrae, and ribs). After multidisciplinary discussion, in December 2022, treatment with lanreotide 60 mg every 28 days was started. It was well tolerated. The dose was increased to 120 mg monthly in April 2023, when the patient came to our attention. MRI in June 2023 confirmed the bone lesions, and 68Ga-DOTATATE PET/CT in July 2023 showed uptake at known sites (stable disease). The patient remained asymptomatic except for malodorous otorrhea and persistent tinnitus. Another 68Ga-DOTATATE PET/CT scan was performed in July 2024 (**Figure 1**), which demonstrated an increase in the expression of somatostatin receptors in the right middle ear and known skeletal sites; therefore, treatment with Denosumab 120 mg every 28 days was started, in addition to lanreotide. The patient is currently being monitored and shows no evidence of disease progression.

## 4 Discussion

We presented three cases of MeNET followed in our Center and collected the 221 published cases, summarized in **Table 1**. They reflected the various clinical scenarios usually associated with this form of neoplasms, varying from localized disease, treated by surgical intervention and possibly radiotherapy, to metastatic disease requiring combined and multidisciplinary therapeutic approaches. This article is focused on well-differentiated NET, characterized by cuboidal or columnar cells with salt and pepper nuclear chromatin, oval nuclei, and eosinophilic cytoplasm in various growth patterns: trabecular, glandular, ribbons, and solid (4).

**Table 1:** Pubmed-published cases of MeNETs fulfilling the inclusion criteria.

Author Year ref	Type	N° of pts	Age	Sex	Symptoms	Other	CT	MRI	Octreoscan PET	Histological pattern	CgA	NSE	CD56	CC/V	Synap	S100	Treatment	Outcome	Follow up
Ketabch S 2001 (21)	CR	1	53	F	I; O; T	itch	+	+	NP	SD, TU-G, TBR	+	+	–	+	–	–	None *	Stability	14 m
Menezes G 2001 (22)	CR, §	1	51	M	NA		NA	NA	NA	NA	+	+	–	+	+	–	S	LR	2 y
Torske KT 2002 (23)	R, §	48	45 (20-80)	21 F 27M	I; O; T		NA	NA	NA	G, TBR, SD	+	–	–	+	–	–	S	40 NED 8 LR	15.7 y
Aquino BF 2002 (24)	CR	1	28	F	I; O	Dizziness	+	NP	NP	G	+	–	–	–	+	–	S	NED	NA
Shibosawa E 2003 (25)	CR	1	64	F	I		+	NP	NP	G, TBR	+	–	–	+	+	–	S	NED	2 Y
Nikanne E 2004 (26)	CR	1	34	F	FP	Otitis	+	+	NP	NA	NA	NA	NA	NA	NA	NA	S	NED	6 y
Chan KC 2005 (27)	CR	1	29	F	I; O; T; FP		+	NP	NP	TBR, G	+	+	–	+	–	–	S	NED	12 m
Ramsey MJ 2005 (28)	R	3	1:29 2:27 3:41	1:M2:F 3:F	I; T		+	+	NP	1: NA 2: TBR, N 3: N, G	1: - 2: + 3: NA	1:+ 2:+ 3: NA	1:- 2:- 3: NA	1:+ 2:+ 3: NA	1:+ 2:+ 3: NA	1:- 2:- 3: NA	S	1: NED 2: MT 3: NED	1: 24 m 2: 48 m 3: 8 m
Isipradit P 2007 (12)	CR	1	32	M	I		+	NP	NP	G, TBR	+	–	–	+	–	–	S	NED	NA
Gaafar A 2008 (19)	CR	1	45	M	I		+	+**	Octreoscan + **	N, R	+	–	+	–	+	–	S + RXT +CT	DOD	11 y
Zehlicke T 2008 (13)	CR	1	53	M	I; FP	dizziness	+	NP	NP	G, TBR, SD	NA	NA	NA	NA	NA	NA	S	NED	24 m
Mori E 2009 (29)	CR	1	29	F	O; T; FP; OR		+	+	NP	SD, G, TBR	+	–	–	+	–	–	S	NED	2 m
Sahan M. 2008 (30)	CR	1	34	M	I; T		+	NP	Octreoscan +** PET **	SD, CRI	+	+	–	–	+	–	S	NED	8 m
Angouridakis N 2009 (31)	CR, §	1	52	F	I; T		NA	NA	NA	CRI	+	+	–	+	+	–	S	NA	NA
Zan E 2009 (32)	CR	1	48	M	I; A; T		+	+	+	G	NA	NA	NA	NA	NA	NA	S	NA	NA
Saliba I 2009 (33)	R	1	32	M	I; O; T; FP		+	NP	NP	G	+	+	–	–	+	–	S	NED	2 y
Aoki M 2010 (34)	CR	1	59	M	I; O	Bleeding	+	+	NP	G; TU	+	–	+	+	+	–	S	NA	NA
Tomazic PV 2011 (35)	CR, §	1	23	M	I; O; A; OR	Dizziness	+	+	NP	TBR, G	NA	NA	NA	NA	NA	NA	S	NA	NA
Verhage-Damen 2011 (36)	CR	1	63	F	T		+	NP	Octreoscan + **	G, TBR	+	–	–	–	+	–	S	NED	NA
Baig S 2012 (37)	CR, §	1	NA	F	I	Bleeding	+	NP	NP	NA	NA	NA	NA	NA	NA	NA	S	NA	NA
Duderstadt M 2012 (38)	R	5	37 (34-61)	4 F 1 M	5 I; 1 T		NA	NA	NA	G, SD, TU	+	+	–	–	+	–	5 S	1 LR 4 NED	2–13 y
Lott Limbach AA 2012 (39)	R	11	36.2	5 M 6 F	T; O	Dizziness	NA	NA	NA	TBR, G, CRI	–	–	–	+	–	–	NA	NA	NA
Shim MJ 2012 (40)	CR	1	17	M	I; T; OR	Bleeding	+	NP	NP	G	–	–	+	+	+	–	S	NED	2 y
Cruz Toro P 2012 (41)	CR	1	54	F	I; A; T		+	+	NP	G, TBR	–	–	–	+	+	–	S	NED	2 y
Fundakowski CE 2013 (42)	CR	1	52	M	I; T		+	NP	PET **	NA	+	–	+	–	+	–	S + RXT	LR+M	10 y
Salzaman R 2012 (43)	CR	1	72	F	I; OR	facial weakness	+	NP	NP	TBR, G	+	–	–	+	+	–	S + RXT	LR+M	8 y
Bittencourt AG 2013 (44)	R	2	1: 41 2: 41	1: F 2: M	I; A; O; T		+	+	NP	SD, TBR, PY	+	–	–	+	+	–	1: S 2: S	1: NED 2: NA	1: 18 m 2: NA
Kim YH 2014 (45)	CR	1	27	F	I; A		+	NP	NP	TBR	+	+	+	+	+	–	S	NED	30 m
Gobel Y 2014 (46)	CR	1	27	M	I		+	NP	NP	NA	+	–	–	–	+	–	S	LR	3 y
Almuhanna K 2014 (47)	CR	1	49	M	I; F		+	NP	NP	TBR	+	–	–	+	+	–	S	NED	10 m
Liu G 2014 (48)	R	1	37	M	I; T; OR		+	NP	NP	G, N, CO	–	–	+	–	+	–	S	LR	8 m
Baku M 2014 (49)	CR	1	48	F	I; A		+	NP	NP	G, SD	+	–	–	–	+	–	S	NED	5 y
Isenring D 2014 (9)	CR	1	52	M	I; A		NP	+	NP	G	+	–	–	–	+	–	S	NED	1 y
Agaimy A 2015 (50)	CR	6	38 (32-53)	4 M 2 F	I; A; T	Otitis/dizziness	+	NP	NP	SD-TBR, TU, CRI, P	+	–	+	+	+	–	S	2 LR 2 NED 2 NA	4.5–22 y
Hu H 2016 (51)	R	1	39	F	I		+	NP	NP	NA	–	+	–	+	+	–	S	NA	NA
Hasan Z 2016 (52)	CR	1	33	M	T; O; FP		NA	NA	NA	TBR	+	–	+	+	+	–	S	NA	NA
Bell D 2017 (18)	CR	10	43 (29-65)	5 F 5M	I; T; O	ear pressure and drainage	+	+	NP	TBR, G, P,	+	+	–	+	+	–	S + RXT + CT	4 NED; 1 LR; 1 M; 4 NA	8–129 m
Wang L 2017 (53)	R	1	39	M	I	dizziness	+	+	NP	SD, G, TBR	+	–	+	+	+	–	S	NED	45 m
Qian ZJ 2017 (54)	CR	1	22	M			NP	+	NP	TBR	–	–	+	+	+	–	S	NA	NA
McCrary H C 2017 (55)	CR	1	38	F	I; O; A		NA	NA	NA	NA	+	–	+	+	+	–	S	NED	Several y
Marinelli JP 2018 (14)	R	32	NA	NA	I; A; O; F; OR; FP	Dizziness	22 +	12 +	2 Octeotride scintigrapy	TBR, N	–	–	–	+	+	–	S	12 LR 20 NED	24–84 m
Sato K 2018 (10)	CR	1	22	F	I; O		NP	+	NP	N	–	–	–	+	+	–	S	NED	11 m
Vilain J 2018 (56)	CR	1	26	F	I; O; A		+	NP	SPECT/CT **	P	+	+	–	+	–	–	S	NED	10 m
He S 2019 (57)	CR	2	1: 60 2: 48	1: M 2: M	I; A; T; OR		+	NP	NP	G, TBR	+	–	–	+	+	–	S	NA	NA
Zaman SU 2019 (58)	CR	1	50	M	I; O; A; FP		+	NP	NP	N	–	–	–	+	+	–	S	NED	3 y
Khaund G 2019 (59)	CR	1	50	M	I; O		+	NP	NP	SD, CBR	+	–	–	+	+	–	S	NA	NA
Federova K 2021 (60)	CR	1	58	F	OR		+	NP	NP	SD, G	+	–	–	–	+	–	S	NED	2 y
Yang L 2020 (61)	CR	1	48	M	I; FP	Recurrent otitis	+	+	NP	TBR	+	–	+	–	+	–	S	NED	NA
Shishido T 2022 (62)	R	1	52	M	I; A; FP		+	+	NP	SD	+	–	–	–	+	–	S	NE	3 m
Bruschini L 2020 (63)	CR	1	40	F	I; A; O	Headache, itching, postural instability	+	+	NP	N, TBR, G	+	–	–	+	+	–	S	NED	12 m
Pontico M 2020 (64)	CR	1	40	F	I; A; O	dizziness	+	+	+Octreoscan/PET	NA	+	–	–	–	+	–	NA	NA	NA
Zwierz A 2021 (65)	R	1	40	M	I; A; F		+	+	NP	C	+	–	–	–	+	–	S	NA	NA
Alciato L 2021 (66)	CR	5	41 (33-49)	3 M 2 F	I; F; FP		+	+	NP	TBR, G	+	–	–	+	+	–	S + RT	2 LR 3 NED	9–129 m
Van der Lans R 2021 (15)	R	9	36 (24-60)	3 M 6 F	I; A; O; T; FP; OR	dizziness	+	+	SPECT + PET +	NA	+	–	–	–	+	–	S RT SSA PRRT	5 LR 2 NED 1 MT 1 None*	21–381 m
Zagaria A 2021 (11)	CR	1	37	M	I; A		NP	+	NP	SD, N, G	+	–	–	–	+	–	S	NED	NA
Lima Ferreira J 2021 (16)	CR	2	1: 38 2:57	1: F 2:M	O; FP		+	+	Ga-DOTA **	NA	NA	NA	NA	NA	NA	NA	S + EBRT+ SSA+ PRRT+ CT	1 DOD; 2 MT	1: 30 m 2: 26 m
Bou Rahal P 2021 (67)	CR	4	1:572:363:414:38	1:M 2:F 3:F 4:M	I; A	Dizziness, otitis	+	+	1 Ga-DOTATATE-PET/CT **	1: TBR-G 3: NA	+	–	–	+	+	–	S	3 NED 1 LR	3–7 y
Chang H 2022 (68)	R	1	70	F	I; O; FP	Ataxia	+	+	NP	SD	–	–	–	+	+	–	S	NED	14 m
Xie B 2022 (69)	CR	5	43.5 (30-62)	2 M 3 F	I; A; T; OR		+	+	NP	NA	–	+	+	+	+	–	S	5 NED	8–148 m
Lachkar A 2022 (70)	CR	1	35	M	I; A		+	NP	NP	G	+	–	+	–	+	–	S	NA	NA
Nabeel M 2023 (71)	CR	1	29	M	I		+	+	PET**	NA	+	–	–	+	+	–	S	NED	NA
Marini K 2023 (72)	CR	1	43	M	I; F		+	NP	Octreoscan **	G	–	–	–	+	+	–	S	NED	12 m
Sukumaran Y 2023 (73)	CR	1	40	M	I; O; OR		+	NP	NP	CRI, G	–	–	+	–	–	–	S	NED	NA
Kvascevicius L 2023 (74)	CR	1	35	F	I		+	+**	NP	SD	+	–	–	+	+	–	S	LR	13 y
Ozdemir O 2023 (75)	CR	1	38	F	I; A		+	+	NP	SD	+	–	+	+	+	–	S	NED	1 y
Sato MP 2023 (76)	CR	1	35	F	NA		+	+	NP	TBR, R	–	–	–	+	+	–	S	MT	15 y
Tsetsos N 2023 (77)	CR	1	45	M	I; A; F; OR		+	+	NP	N, TBR	+	–	–	+	+	–	S	NED	16 m
Niewiadomska A 2024 (78)	CR	1	39	F	I		+	+	NP	N	+	–	+	+	–	–	S	NED	NA
Vrebac I 2024 (79)	CR	1	60	M	I		+	+	NP	NA	–	–	+	–	+	–	S	NED	20 m
Alharbi N 2024 (80)	CR	1	46	M	I; OR		+	+	NP	NA	+	–	+	–	+	–	S	MT?	4 m
You D 2024 (81)	R	10	39.3	5M; 5 F	I; A; O; F; FP	Dizziness	+	+	NP	NA	–	–	+	+	+	–	S	NED	7–84 m
Ferney A 2024 (82)	CR	1	21	M	I; O; FP		+	+	NP	TBR, TU	+	–	–	+	+	–	S	NED	1 y
Sun Y 2024 (20)	R	1	29	M	I; FP		+	+ **	NP	G	–	–	+	–	+	–	S + CT	DOD	6 y
Zeng N 2025 (83)	CR	1	27	M	I; A; T		+	+	NP	N, G, SD	+	–	–	+	+	–	S	NED	6 m
Yamamoto H 2025 (84)	CR	2	40	F	I		+	+	NP	NA	–	–	+	–	+	–	S	NED	1: 4y 2: 3 y
Tsiouvaka 2025 (85)	CR	1	38	M	I; A; T		+	+	NP	NA	–	–	–	+	+	–	S	NED	4 y
Schmitz L 2025 (86)	CR	1	65	F	I		+	+	+	NA	–	–	–	+	+	–	S + RXT + SSA	MT	NA
Engel MSD 2025 (17)	CR	3	1:362:533:33	1:F 2:M 3:M	FP; O; I; T	Otitis	1: + 2: + 3: +	1: + 2: + 3: +	1: + 2: NP 3: +	1: CRI 2: TBR, N 3: NA	1:+ 2: NA 3: NA	1:- 2: NA 3: NA	1:- 2: NA 3: NA	1:- 2: NA 3: NA	1:+ 2: NA 3: NA	1:- 2: NA 3: NA	1: S + RXT +PRRT 2: S+ RXT 3: S +RXT +SSA+ PRRT	1, 2 DOD 3 MT	1: 11 y 2: 9 y 3: 11 y
Shin SH 2025 (87)	CR	1	19	F	FP; I	Otitis	+	+	NP	TU	–	–	+	–	+	–	S	NED	2 y

CR, case report; R, review; §, The full text is not available, only the abstract can be consulted; pts, patients; I, ipoacusia; T, tinnitus; O, Otalgia; A, Aural Fullness; FP, facial paralysis; OR Otorrhea NA, not available; NP, not performed (declair by authors); CC, cytokeratines; V, vimentine; RXT, Radiation X-ray Therapy; CT, chemiotherapy; SA, somatostatin analogues; RLT, radioligand therapy; NED, no evidence of disease; LR, local recurrence; MT, metastatic tumor; m, months; y, years; S, surgery; DOD, dead of disease; SD, solid; TBR, trabecular; G, glandular; TU, tubular; CRI, cribiform; P, plasmacytoid; N, nested; R, ribboned; PY, papillary; CO, cords; M, mucinous. *The patient refused surgical treatment. ** Test performed during follow-up.

The principal differential diagnosis of well-differentiated middle ear neuroendocrine tumor (MeNET) is paraganglioma. Although both tumors express neuroendocrine markers (synaptophysin, chromogranin A), MeNET shows epithelial differentiation with diffuse positivity for broad-spectrum cytokeratins (CAM5.2, AE1/AE3) (7). In contrast, paraganglioma is a non-epithelial neoplasm, lacks cytokeratin expression, and typically demonstrates a S100 positivity. Therefore, cytokeratin expression represents the key discriminating feature. CK20 negativity futher supports the exclusion of Merkel cell carcinoma (8). Accurate immunohistochemical profiling is therefore essential to establish the correct diagnosis and guide management.

The clinical presentation can be silent (as in case 1), but it is usually characterized by local symptoms, most frequently ipoacusia (90.9%), followed by tinnitus (39.9%), aural fullness (33.5%), otalgia (31.7%), facial paralysis (27.6%), otorrhea (16.7%) and dizziness (14.0%). Less frequently observed symptoms include: otitis (5.9%), bleeding (3.6%), itch (2.3%), ear pressure and drainage, facial weakness, headache, and postural instability and ataxia (1.3% each).

Based on the reviewed literature, MeNETs are reported as sporadic and solitary tumors. None of the analyzed cases explicitly documented an association with multiple endocrine neoplasia syndromes, including MEN1. Genetic analyses were not performed, and no recurrent germline or somatic mutations have been consistently reported to date. In our cases, we did not perform genetic testing because the patients did not have cafe-au-lait spots or cutaneous neurofibromas (excluding neurofibromatosis type 1) and did not presented pituitary alterations or hyperparathyroidism, in accordance with the latest available guidelines that recommended screening for MEN 1 only if in case of at least 2 of the 3 main conditions associated with this syndrome or in patients younger than 40 years old with primary hyperparathyroidism (6).

Morphological imaging with CT was the most used diagnostic technique, performed in all but three cases (9–11), while, interestingly, MRI was performed in fewer cases and was negative in two studies (12, 13). At CT, MeNETs appeared as a hypodense mass encased in the ossicles, which more often are not destroyed by the mass, while ossicular erosion is less common. CT is usually the first test performed in case of symptoms such as hearing loss with a negative otoscopic examination. CT scans can identify the presence of lesions in the tympanic cavity or erosion or extension to adjacent structures, but CT is not specific for neuroendocrine tumours detection. At MRI, MeNET showed hyperintensity in T1-weighted sequences and hypointensity in T2-weighted sequences, with contrast-enhancement and no restriction in DWI (Diffusion-Weighted Imaging) (14). Contrast-enhanced MRI defines the extent of the tumour and involvement of adjacent soft tissues better than CT. This is particularly useful for surgical planning.

While CT and MRI describe the anatomical extent of the lesion, nuclear medicine imaging plays a role in the functional assessment of these tumours. Many neuroendocrine neoplasms express somatostatin receptors, allowing targeted imaging with radiolabelled peptides such as 68Ga-DOTATATE PET/CT, confirming the neuroendocrine nature of the lesion. From the literature review emerges that, in many cases, functional imaging was not performed, primarily because the diagnosis was made after radical surgery. When performed, functional imaging (with both Octreoscan and PET/CT with Gallium peptides) was able to detect the disease. In our experience, functional imaging with 68Ga-DOTATATE PET/CT is effective in the detection of both the primary mass and the possible metastases, with the highest accuracy. Even if no data are available on the possible use of Artificial Intelligence in MeNETs, that could be an opportunity to improve pre-surgical diagnosis, by multimodal imaging integration.

In localized disease, surgical intervention is the treatment of choice. According to the literature, 183 patients underwent surgery, together with radiotherapy in 34 cases. With the exception of missing data, all other patients refused surgery for personal choice. In 42 cases, surgery was not curative, and patients experienced local relapse. In these cases, new surgical intervention or radiotherapy was used as first therapeutic strategy. In inoperable cases or in case of metastatic disease, medical treatment is required. Medical treatment, mainly somatostatin analogs, was used in 28 patients and was able to maintain stability, as in our case 3. Only three articles describe the use of radioligand therapy in patients with metastatic disease (15–17). The presence of metastases, somatostatin receptor expression, tumor burden, grading, and symptom control are key factors guiding the use of somatostatin analogues and, potentially, peptide receptor radionuclide therapy.

In this field of well-differentiated NET, chemotherapy has a limited role, and can be considered in case of rapidly progressive disease, high tumor burden, and a lack of alternative therapeutic strategies. Chemotherapy has been used in one case as adjuvant therapy after laminectomy for cervical metastases (no protocol described) (18), in three cases for metastatic diseases. In two cases, carboplatin and etoposide was administered (with 15 (16) and 5 (19) months of survival), in another case a combination of cisplatin, temozolomide and anlotinib was used, and the patient died 10 months after last surgical intervention (20).

Five patients died from the disease, 30 months (16), 6 years (20), 9 years (17) and 11 years (17, 19) after diagnosis.

The available evidence suggests that prognostic factors in MeNETs consist of tumor size, extent of surgical resection, bone invasion, and presence of metastatic disease, whereas the Ki-67 LI tends to be low and therefore its impact on prognosis is less evident. Coherently, treatment strategy is less impacted by Ki-67 LI, since most of the cases are low-grade tumors, managed as grade 1 or grade 2 with low Ki-67 LI NETs of other sites.

Recurrences are mainly localized and can occur years after the first treatment, emphasizing the importance of extended monitoring. A long-term follow-up is strongly recommended, particularly a long surveillance extending beyond 10 years. Even if a standardized monitoring protocol does not exist, it is possible to suggest a clinical and radiological follow-up with periodic otoscopic examination, audiological assessment, imaging surveillance (preferably using MRI), and functional imaging in selected cases with suspected recurrence or metastatic disease.

It is important to highlight that diagnostic and treatment suggestions only came from case reports and case series articles, given the rarity of MeNETs. Considering the low quality of evidence available, MeNETs management lacks site-specific guidelines and clinical approach is derived from other NET types, highlighting the necessity for a unified classification and multidisciplinary approach. It is mandatory to manage patients in cancer centers with a dedicated multidisciplinary tumor board.

## 5 Conclusions

MeNET are rare neuroendocrine tumors. Despite often being localized and therefore potentially curable with surgery, they may be characterized by local recurrence and metastatic spread. In this context, the available therapeutic strategies are limited and borrowed from gastro-entero-pancreatic or pulmonary NETs. Further studies on natural history and response to standard treatment are warranted. Histopathological diagnosis is essential to guide treatment and should be in accordance with the WHO 2022 classification of head and neck neuroendocrine tumors. The use of the same classifications will also contribute to research in this field.

## Data Availability

The datasets presented in this study can be found in online repositories. The names of the repository/repositories and accession number(s) can be found below: https://gbox.garr.it/it/.
